# Hydrogen Peroxide, Signaling in Disguise during Metal Phytotoxicity

**DOI:** 10.3389/fpls.2016.00470

**Published:** 2016-04-25

**Authors:** Ann Cuypers, Sophie Hendrix, Rafaela Amaral dos Reis, Stefanie De Smet, Jana Deckers, Heidi Gielen, Marijke Jozefczak, Christophe Loix, Hanne Vercampt, Jaco Vangronsveld, Els Keunen

**Affiliations:** Environmental Biology, Centre for Environmental Sciences, Hasselt UniversityDiepenbeek, Belgium

**Keywords:** metals, hydrogen peroxide, oxidative stress, damage, signaling, crosstalk

## Abstract

Plants exposed to excess metals are challenged by an increased generation of reactive oxygen species (ROS) such as superoxide (${\text{O}}_{2}^{•-}$), hydrogen peroxide (H_2_O_2_) and the hydroxyl radical (^•^OH). The mechanisms underlying this oxidative challenge are often dependent on metal-specific properties and might play a role in stress perception, signaling and acclimation. Although ROS were initially considered as toxic compounds causing damage to various cellular structures, their role as signaling molecules became a topic of intense research over the last decade. Hydrogen peroxide in particular is important in signaling because of its relatively low toxicity, long lifespan and its ability to cross cellular membranes. The delicate balance between its production and scavenging by a plethora of enzymatic and metabolic antioxidants is crucial in the onset of diverse signaling cascades that finally lead to plant acclimation to metal stress. In this review, our current knowledge on the dual role of ROS in metal-exposed plants is presented. Evidence for a relationship between H_2_O_2_ and plant metal tolerance is provided. Furthermore, emphasis is put on recent advances in understanding cellular damage and downstream signaling responses as a result of metal-induced H_2_O_2_ production. Finally, special attention is paid to the interaction between H_2_O_2_ and other signaling components such as transcription factors, mitogen-activated protein kinases, phytohormones and regulating systems (e.g. microRNAs). These responses potentially underlie metal-induced senescence in plants. Elucidating the signaling network activated during metal stress is a pivotal step to make progress in applied technologies like phytoremediation of polluted soils.

## The relationship between metals and oxidative stress in plants

Pollution of soils, air, (ground)water and sediments with toxic metals is one of the major problems our industrialized world is currently facing. Naturally occurring levels of these metals have been significantly exceeded by anthropogenic activities over the past two centuries. Mining and industry, as well as the use of phosphate fertilizers and sewage sludge in agriculture have jointly contributed to an increased production and emission of metals. As opposed to many organic contaminants, metals are non-biodegradable, resulting in their extended persistence in the environment. In addition, food and feed crop plants facilitate the entry of toxic metals into the food chain, thereby leading to bio-enrichment and enhanced risks for human health (Cuypers et al., 2009; Sharma and Dietz, 2009). The latter has been demonstrated by a plethora of *in vitro, in vivo* and epidemiological studies, revealing that the highest health risks are associated with exposure to cadmium (Cd), lead (Pb) and mercury (Hg). Adverse metal-induced health effects are wide-ranging, for example with kidney damage, bone effects and cancer related to human Cd exposure (Järup, 2003; Nair et al., 2013). Nevertheless, metal exposure persists and even increases in less developed countries (Järup, 2003), urging the need to remediate metal-polluted soils.

Metals are categorized as essential or non-essential for plant growth, with different dose-response curves for both classes (Lin and Aarts, 2012). Essential micronutrients such as copper (Cu), iron (Fe), nickel (Ni) and zinc (Zn) function as cofactors in over 1500 proteins crucial for the plant's metabolism. For example, Cu is cardinal for photosynthesis and mitochondrial respiration, while Zn-containing enzymes are important regulators of transcription and translation. For that reason, either too low or high levels of these essential metals would adversely affect plant growth and development (Hänsch and Mendel, 2009; Pilon et al., 2009). To avoid both deficiency and excess, plant cells possess different mechanisms to tightly control the concentrations of essential metals (Lin and Aarts, 2012). However, even low concentrations of non-essential metals such as Cd, Pb and Hg disturb biochemical and physiological processes and decrease plant productivity (Lin and Aarts, 2012).

**Figure 1:**
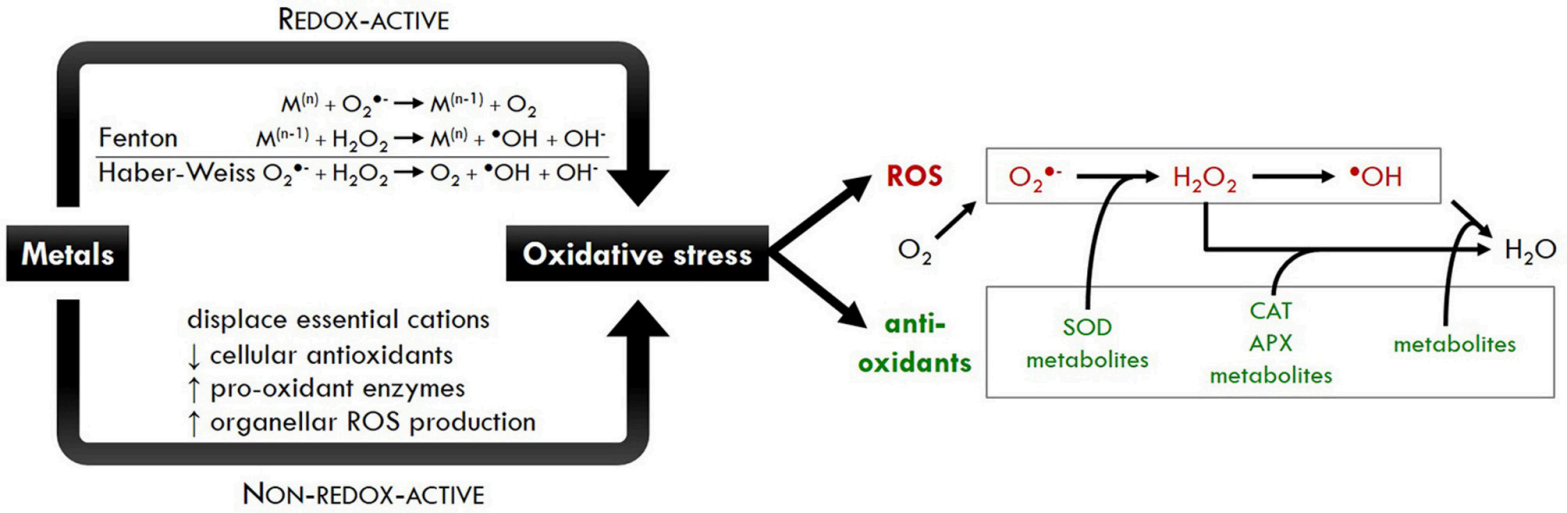
**Schematic overview of metal-induced oxidative stress**. Redox-active metals such as Cu and Fe can participate in the Fenton and Haber-Weiss reactions, finally leading to the formation of highly toxic ^•^OH radicals from H_2_O_2_. On the other hand, non-redox-active metals such as Cd and Zn can only indirectly contribute to ROS production by (1) displacing essential cations, (2) depleting cellular antioxidants, (3) increasing the activity of ROS producing enzymes and/or (4) enhancing ROS production in organelles. The net result for both classes of metals is the induction of oxidative stress, an imbalance between ROS and antioxidants in favor of the former. Abbreviations: APX, ascorbate peroxidase; CAT, catalase; H_2_O_2_, hydrogen peroxide; M^(n)^, oxidized redox-active metal; M^(n−1)^, reduced redox-active metal; ${\text{O}}_{2}^{•-}$, superoxide; OH^−^, hydroxide ion; ^•^OH, hydroxyl radical; SOD, superoxide dismutase.

Sharma and Dietz (2009) have described three major mechanisms underlying metal toxicity in plants. First, different metals show a high affinity toward sulfur or nitrogen donors within proteins, which might interfere with cellular metabolism. Metals are also able to displace essential cations from their specific binding sites within an enzyme. For example, Cd^2+^ was suggested to competitively bind to the essential calcium (Ca^2+^) site in photosystem II during photoactivation (Faller et al., 2005). Finally, multiple studies have demonstrated that exposure of plants to a diverse array of metals elicits oxidative stress, indicating a misbalance between the production and neutralization of reactive oxygen species (ROS) such as superoxide (${\text{O}}_{2}^{•-}$), hydrogen peroxide (H_2_O_2_) and the hydroxyl radical (^•^OH) (Schützendübel and Polle, 2002; Sharma and Dietz, 2009). In view of the different chemical properties of metals, two modes of action can be distinguished. Under physiological conditions, redox-active metals such as Cu and Fe exist in different oxidation states (Cu^+∕2+^ and Fe^2+∕3+^). This enables both metals to directly participate in the Fenton and Haber-Weiss reactions, finally leading to the formation of highly toxic ^•^OH radicals from H_2_O_2_ (Figure 1; Schützendübel and Polle, 2002; Hänsch and Mendel, 2009; Sharma and Dietz, 2009). On the other hand, physiologically non-redox-active metals such as Cd, Hg, and Zn only indirectly contribute to increased ROS production, for example by depleting or inhibiting cellular antioxidants (Figure 1; Schützendübel and Polle, 2002; Sharma and Dietz, 2009).

**Figure 2:**
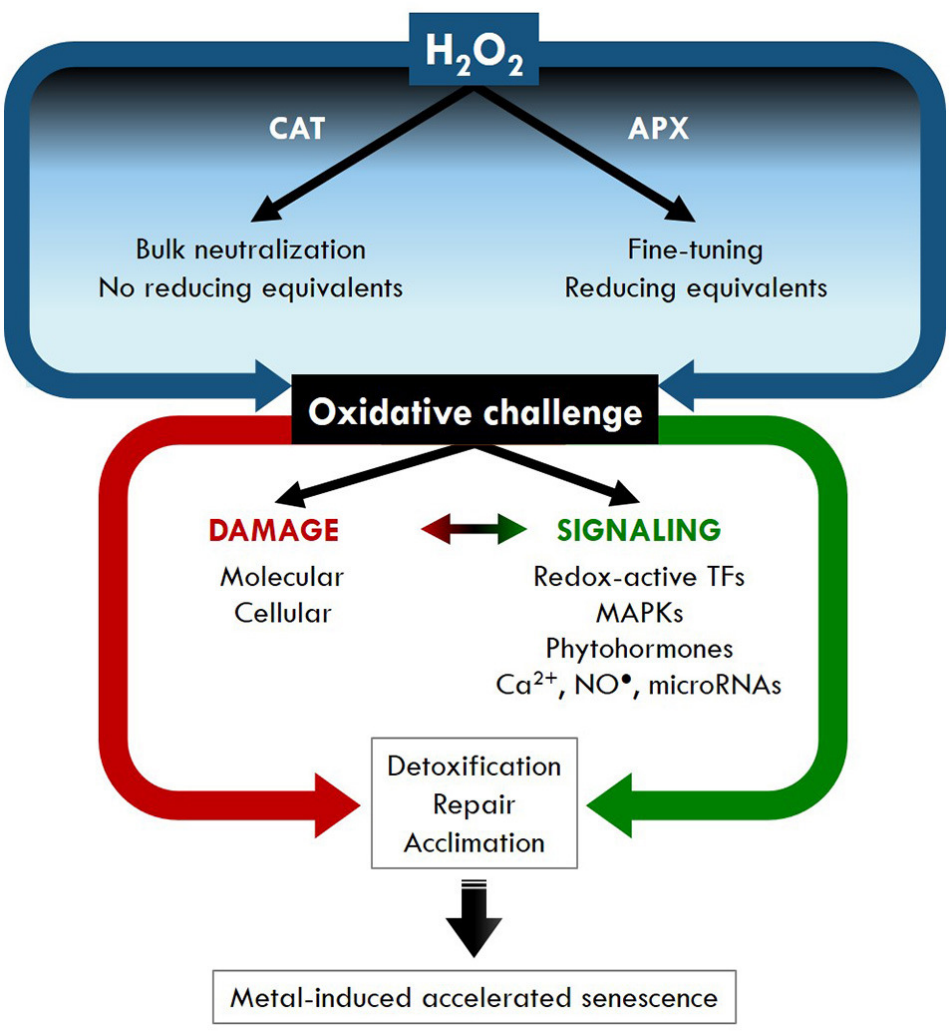
**Generalized model for the central role of hydrogen peroxide (H_**2**_O_**2**_) in metal-induced oxidative damage vs. signaling responses**. Ascorbate peroxidase (APX) and catalase (CAT) are the two most important enzymes that counterbalance metal-induced H_2_O_2_ production in plants. However, APX has a much higher affinity for H_2_O_2_ as compared to CAT. Therefore, the former enzyme is considered to be primarily involved in the fine-tuning of H_2_O_2_ levels crucial for their signaling function. Conversely, CAT is important for the bulk removal of excess H_2_O_2_ produced in stressed plants. In addition, while APX uses AsA as a reducing agent to detoxify H_2_O_2_, CAT does not need any reducing equivalents. Depending on its extent, the metal-induced rise in H_2_O_2_ content can lead to molecular and cellular damage and/or signaling. Different studies highlight the interaction between ROS/H_2_O_2_ and signaling components such as redox-active transcription factors (TFs), mitogen-activated protein kinases (MAPKs), phytohormones, Ca^2+^, NO^•^ and regulating systems like miRNAs. Finally, detoxification, repair and acclimation responses are activated, with accelerated senescence as a potential last resort in metal-exposed plants.

The term “oxidative stress” implies a harmful process, which is mainly related to the oxidizing nature of ROS. However, intense research over the past decades has shifted this paradigm, pointing toward a dual role for ROS as damaging vs. signaling compounds (Foyer and Noctor, 2005). Currently, ROS and H_2_O_2_ in particular are considered as essential components of signal transduction used by plants to respond to developmental and environmental cues. In this review, it is our intent to provide an overview of the experimental evidence underlying a dual role for H_2_O_2_ during metal stress in plants. Within this framework, both H_2_O_2_-induced damage and signaling—including its targets and interaction with other signaling pathways and regulating systems—are highlighted. Ultimately, the term “oxidative challenge” is preferred, as this implies the harmful vs. beneficial effects of H_2_O_2_ produced in metal-exposed plants (Figure 2).

## Hydrogen peroxide, a signaling molecule in disguise

Both energy transfer to as well as incomplete reduction of O_2_ generate ROS such as singlet oxygen (^1^O_2_) and ${\text{O}}_{2}^{•-}$, H_2_O_2_ and ^•^OH respectively. These reactive intermediates are byproducts of physiological processes such as photosynthesis and respiration, with a high oxidizing potential toward DNA, lipids and proteins. However, not all ROS are equally reactive, with ${\text{O}}_{2}^{•-}$ and H_2_O_2_ being rather selective in their reactions and ^•^OH attacking all molecules in its surroundings (Halliwell, 2006; Møller et al., 2007). Under steady-state conditions, antioxidant enzymes and metabolites tightly control ROS concentrations in different cellular compartments to prevent oxidative damage (Mittler et al., 2004, 2011). In addition, plants have developed a way to employ low levels of ROS as signaling compounds to appropriately and coordinately respond to developmental as well as environmental cues (Petrov and Van Breusegem, 2012). It has long been known that different biotic (e.g. pathogen attack) and abiotic (e.g. drought, salinity, heat and metal stress) stimuli increase ROS generation in plants, leading to a misbalance between ROS and antioxidants in favor of the former (Dat et al., 2000; Apel and Hirt, 2004). Especially under these conditions, the use of ROS in signal transduction can contribute to acclimation and eventually tolerance to various stressors (Hossain et al., 2015).

Among all ROS, H_2_O_2_ is often put forward as the most attractive signaling molecule (Neill S. et al., 2002; Neill S. J. et al., 2002; Foyer and Noctor, 2005; Petrov and Van Breusegem, 2012). It is produced by a two-step reduction of molecular O_2_. Superoxide—generated after the first reduction step—is converted into H_2_O_2_, for example by superoxide dismutase (SOD). Subsequently, H_2_O_2_ can give rise to highly toxic ^•^OH radicals through the Fenton and Haber-Weiss reactions with the help of free redox-active metal ions (Figure 1; Halliwell, 2006). With a half-life of 1 ms, H_2_O_2_ is relatively stable as compared to ${\text{O}}_{2}^{•-}$ and ^•^OH that have a half-life of only 1 μs and 1 ns, respectively (Møller et al., 2007). Additional advantages are its high cellular abundance (up to the low millimolar range) (Cheeseman, 2006; Møller et al., 2007), its small size (Petrov and Van Breusegem, 2012) and its ability to cross cellular membranes through aquaporins and thereby migrate to different cellular compartments (Bienert et al., 2006, 2007; Bienert and Chaumont, 2014). Furthermore, H_2_O_2_ is an uncharged non-radical with an intermediate oxidation number (−1 for each oxygen atom), implying both oxidizing and reducing properties (Bienert et al., 2007; Bienert and Chaumont, 2014). With regard to H_2_O_2_ scavenging, it is important to keep in mind the unique property of catalase (CAT) among all antioxidative enzymes: it is able to convert H_2_O_2_ to H_2_O_2_ and O_2_ without the use of reducing equivalents (see Section “Production and Scavenging of H_2_O_2_ in Metal-Exposed Plants”) (Bienert et al., 2007; Das and Roychoudhury, 2014). The fact that H_2_O_2_ scavenging by CAT occurs in an energy-efficient way can be a crucial asset under environmental stress conditions, when energy is required to set up an appropriate defense response (Gechev et al., 2006; Das and Roychoudhury, 2014).

Reactive oxygen species are able to transmit a signal by oxidizing a target molecule, for example a transcription factor (Mittler et al., 2004). The relatively long-living H_2_O_2_ can travel a cellular distance up to 1 μm and brings the signal close to its target, thereby acting as primary messenger. However, the cellular distance traveled by more short-living ROS ranges from a mere nm (^•^OH) up to 30 nm (^1^O_2_ and ${\text{O}}_{2}^{•-}$). These will therefore react with a cellular compound close to their production site, with the oxidation product acting as second messenger (Møller et al., 2007). However, both routes lead to the same net signaling result for ROS with different physicochemical properties. In the following paragraphs, the production and scavenging of H_2_O_2_ is discussed in the light of metal stress. Furthermore, results from priming experiments and screenings of metal tolerant vs. sensitive genotypes/ecotypes have revealed a strong relationship between H_2_O_2_ and metal tolerance in plants.

### Production and scavenging of H_2_O_2_ in metal-exposed plants

In plants, H_2_O_2_ and other ROS are continuously produced in different subcellular compartments as byproducts of various metabolic reactions. While most ROS in plant cells originate from chloroplasts and peroxisomes, mitochondria are the most important ROS producers under dark conditions and in non-photosynthetic tissues (Navrot et al., 2007; Das and Roychoudhury, 2014). In chloroplasts and mitochondria, leakage of electrons to O_2_ as a consequence of electron transport chain over reduction can generate ${\text{O}}_{2}^{•-}$ radicals, which can subsequently be converted to H_2_O_2_. In peroxisomes, H_2_O_2_ can be directly produced by oxidation reactions of fatty acids and glycolate formed during photorespiration (Petrov and Van Breusegem, 2012).

On the other hand, ROS can also be enzymatically generated in the apoplast. At the plasma membrane, ${\text{O}}_{2}^{•-}$ is generated by NADPH oxidases. These enzymes are homologs of the mammalian respiratory burst oxidase gp91^phox^ and are therefore referred to as respiratory burst oxidase homologs (RBOHs) (O'Brien et al., 2012). Using NADPH as a cytosolic electron donor, they catalyze the reduction of apoplastic O_2_ to ${\text{O}}_{2}^{•-}$, which can then be dismutated to H_2_O_2_ either non-enzymatically or by the action of SOD. Furthermore, apoplastic ROS can also be produced by cell wall-anchored class III peroxidases. Although these enzymes are also involved in H_2_O_2_ scavenging, they are able to generate H_2_O_2_ in the presence of a strong reductant. Reactive oxygen species produced by the action of these peroxidases play an important role in several developmental processes including cell wall cross-linking and loosening (O'Brien et al., 2012; Kärkönen and Kuchitsu, 2015).

While ROS production in organelles and the apoplast continuously occurs under physiological growth conditions, it can be greatly enhanced by biotic and abiotic stress factors (Gechev et al., 2006; Petrov and Van Breusegem, 2012). As demonstrated in Table 1, exposure to even environmentally relevant metal concentrations increased the production of H_2_O_2_ in a wide variety of plant species. As discussed before, the mechanisms underlying metal-induced ROS production in plants are dependent on the chemical properties of the metal. Indirect metal-induced ROS production can be achieved by several mechanisms (Cuypers et al., 2012). Metals can for example inhibit the activity of various enzymes by binding to their functional groups or by displacement of essential cations in specific binding sites (Gupta et al., 2009; Cuypers et al., 2011). In this way, they can disturb the action of enzymes involved in antioxidative defense and physiological processes such as respiration and photosynthesis, thereby increasing ROS production. Furthermore, metals are able to deplete the pool of the important antioxidant glutathione (GSH), thereby also disturbing the ROS balance (Lee et al., 2003). In addition, several metals were shown to increase ROS production by plasma membrane-bound NADPH oxidases (Figure 1; Romero-Puertas et al., 2004; Hao et al., 2006; Remans et al., 2010).

**Table 1:** **Metal-induced H_**2**_O_**2**_ production and scavenging in plants**.

**Metal**		**Species**		**H**_**2**_**O**_**2**_ **scavenging**				**References**
				**APX**		**CAT**		
			**H_2_O_2_ production**	**Activity**	**Gene expression**	**Activity**	**Gene expression**	
Essential	Cu	*Arabidopsis thaliana*	x	x	x	x	x	Cuypers et al., 2011
			x					Liu et al., 2015
			x					Martínez-Peñalver et al., 2012
			x		x		x	Opdenakker et al., 2012a
			x					Yuan et al., 2013
		*Cucumis sativus*	x			x		İşeri et al., 2011
		*Hordeum vulgare*	x	x		x		Hu et al., 2015
		*Ipomoea batatas*	x					Kim et al., 2010
		*Matricaria chamomilla*	x					Kováčik et al., 2010b
			x	x				Kováčik et al., 2010a
		*Medicago truncatula*	x					Macovei et al., 2010
		*Nicotiana tabacum*	x	x				Xia et al., 2012
		*Oryza sativa*	x	x		x		Mostofa et al., 2015a
			x	x				Thounaojam et al., 2012
		*Pauwlonia fortunei*	x	x		x		Wang J. et al., 2010
		*Silene dioica*	x	x				Kováčik et al., 2010b
		*Silene vulgaris*	x	x				Kováčik et al., 2010b
		*Solanum lycopersicum^a^*	x	x				İşeri et al., 2011
		*Spirodela polyrhiza*	x	x		x		Upadhyay and Panda, 2010
	Ni	*Brassica juncea*	x	x				Khan and Khan, 2014
		*Brassica napus*	x	x		x		Kazemi et al., 2010
		*Vicia sativa*	x			x		Ivanishchev and Abramova, 2015
	Zn	*Arabidopsis thaliana*	x	x	x	x	x	Remans et al., 2012a
		*Brassica juncea*	x	x				Feigl et al., 2015
			x	x				Khan and Khan, 2014
		*Gossypium hirsutum*	x	x		x		Anwaar et al., 2015
		*Ipomoea batatas*	x					Kim et al., 2010
		*Lactuca sativa*	x					Barrameda-Medina et al., 2014
		*Myracrodruon urundeuva*	x	x		x		Gomes et al., 2013
		*Pauwlonia fortunei*	x	x		x		Wang J. et al., 2010
		*Phaseolus vulgaris*	x					Michael and Krishnaswamy, 2011
		*Populus* × *canescens*	x	x		x		Shi et al., 2015
		*Solanum melongena*	x	x				Wu et al., 2015
		*Solanum nigrum*	x	x	x	x	x	Xu Q. S. et al., 2010
		*Spirodela polyrhiza*	x	x		x		Upadhyay and Panda, 2010
		*Verbacum thapsus*	x	x				Morina et al., 2010
Non-essential	Al	*Cucumis sativus*	x	x		x		Pereira et al., 2010
		*Nicotiana tabacum*	x	x				Yin et al., 2010
Non-essential	Cd	*Arabidopsis thaliana*	x x	x	x		x	Cuypers et al., 2011 Martínez-Peñalver et al., 2012
			x			x		Tao et al., 2013
		*Boehmeria nivea*	x	x				Tang et al., 2015
		*Brassica campestris*	x					Anjum et al., 2014
		*Brassica juncea*	x					Masood et al., 2012
		*Brassica napus*	x	x		x		Ali et al., 2013
		*Citrus paradisi* × *Poncirus trifoliata*	x			x		Podazza et al., 2012
		*Dittrichia viscosa*	x	x		x		Fernández et al., 2013
		*Glycine max*	x	x		x		Pérez-Chaca et al., 2014
		*Helianthus annuus*	x	x		x		Saidi et al., 2014
		*Ipomoea batatas*	x					Kim et al., 2010
		*Kosteletzkya virginica*	x	x		x		Han et al., 2013
		*Lactuca sativa*	x	x		x		Monteiro et al., 2012
		*Lepidium sativum*	x	x		x		Gill et al., 2012
		*Lupinus luteus*	x					Arasimowicz-Jelonek et al., 2012
		*Nicotiana tabacum*	x	x		x		Iannone et al., 2010
		*Oryza sativa*	x	x		x		Chou et al., 2011
			x	x		x		Mostofa et al., 2015b
			x			x		Singh and Shah, 2014
			x			x		Srivastava et al., 2014
			x	x				Srivastava et al., 2015
			x					Wang et al., 2014
			x					Yu et al., 2015
		*Populus cathayana*	x			x		He et al., 2013
		*Populus nigra*	x	x		x		He et al., 2013
		*Populus popularis*	x	x				He et al., 2013
		*Populus* × *canadensis*	x					Di Baccio et al., 2014
		*Populus* × *canescens*	x	x		x		He et al., 2011
		*Sedum alfredii*	x			x		Tian et al., 2011
		*Solanum lycopersicum*	x	x		x		Ahammad et al., 2013
			x	x		x		Monteiro et al., 2011
		*Solanum nigrum*	x	x		x		Deng et al., 2010
			x	x		x		Liu et al., 2013
		*Trigonella foenum-graecum*	x	x		x		Zayneb et al., 2015
		*Triticum aestivum*	x					Moussa and El-Gamal, 2010
		*Vigna radiata*	x					Anjum et al., 2014
		*Zea mays*	x					Wahid and Khaliq, 2015
		*Zygophyllum fabago*	x	x		x		Yildiztugay and Ozfidan-Konakci, 2015
	Hg	*Juncus maritimus*	x	x		x		Anjum et al., 2015
		*Medicago sativa*	x					Montero-Palmero et al., 2014
Non-essential	Pb	*Arabidopsis thaliana*	x x			x		Tao et al., 2013 Yu et al., 2012
		*Atractylodes macrocephala*	x	x		x		Wang et al., 2013
		*Brassica napus*	x	x		x		Ali et al., 2014
		*Hordeum vulgare*	x					Legocka et al., 2015
		*Lemna trisulca*	x					Samardakiewicz et al., 2015
		*Nymphoides peltatum*	x			x		Qiao et al., 2013
		*Oryza sativa*	x			x		Srivastava et al., 2014
		*Pauwlonia fortunei*	x	x				Wang J. et al., 2010
		*Talinum triangulare*	x	x		x		Kumar et al., 2013
		*Triticum aestivum*	x	x		x		Kaur et al., 2013
			x	x		x		Kaur et al., 2015
		*Vicia faba*	x					Shahid et al., 2012
		*Zygophyllum fabago*	x	x		x		López-Orenes et al., 2014

a *In article as Lycopersicum esculentum*.

*Metals have the capacity to induce oxidative stress in plants. An increase in H_2_O_2_ levels is an indicator of the disturbed redox balance. Plant cells have defense mechanisms to scavenge excess ROS, such as the antioxidative enzymes ascorbate peroxidase (APX) and catalase (CAT). The following table catalogs recent research articles (published since 2010) that reported metal-induced H_2_O_2_ production. The effects on APX and CAT, at the level of both gene expression and enzymatic activity, are indexed according to essential (Cu, Ni, and Zn) and non-essential metals (Al, Cd, Hg, and Pb) and plant species*.

In order to prevent cellular damage as a result of increased ROS production, plants possess an extensive antioxidative defense system consisting of both non-enzymatic and enzymatic compounds (Figure 1). Two important non-enzymatic antioxidants are the water-soluble metabolites ascorbate (AsA) and GSH. Ascorbate can directly scavenge ${\text{O}}_{2}^{•-}$, H_2_O_2_, and ^•^OH radicals and is involved in the regeneration of other antioxidants such as α-tocopherol (Das and Roychoudhury, 2014). Furthermore, it plays an important role in the AsA-GSH cycle. In the first step of this cycle, ascorbate peroxidase (APX) detoxifies H_2_O_2_ to H_2_O using AsA as the reducing agent. Subsequently, the reconversion of AsA to its reduced form is coupled to the oxidation of GSH, which is again reduced by the action of glutathione reductase (GR) (Cuypers et al., 2012). In addition to its involvement in the AsA-GSH cycle, GSH can also directly detoxify ROS and is the substrate of glutathione-S-transferase (GST) enzymes, catalyzing the conjugation of GSH with electrophilic compounds. Plant GSTs are subdivided into several classes and are involved in a wide range of functions including the detoxification of xenobiotics (e.g. herbicides) and products of oxidative DNA and lipid damage (Marrs, 1996; Gill and Tuteja, 2010). Furthermore, GSH plays a role in the scavenging of metals via its sulfhydryl group and is also the precursor of metal-chelating phytochelatins (PCs) (Jozefczak et al., 2012; Noctor et al., 2012). In addition to PCs, also metallothioneins (MTs) are able to bind metals such as Cu, Cd and Zn through the thiol groups of their cysteine residues. Furthermore, several studies suggest that MTs are directly involved in ROS scavenging (Hassinen et al., 2011).

In contrast to the water-soluble AsA and GSH, α-tocopherol and carotenoids are important lipid-soluble antioxidative metabolites. They are involved in protecting membranes against lipid peroxidation and preventing damage to the photosynthetic machinery, respectively (Das and Roychoudhury, 2014). In addition, the amino acid proline accumulates in plants under abiotic stress conditions including metal exposure (Sharma and Dietz, 2006). Proline is able to quench ^1^O_2_ and scavenge ^•^OH radicals *in vitro*, and several studies have attributed an antioxidant function to proline under metal stress *in vivo* as well (Sharma and Dietz, 2006). For example, pretreatment of *Oryza sativa* plants with proline decreased the accumulation of H_2_O_2_ and lipid peroxidation after Hg exposure (Wang et al., 2009). These observations might be related to the fact that proline is able to protect and stabilize ROS scavenging enzymes such as CAT and peroxidases (Sharma and Dietz, 2006; Szabados and Savouré, 2009).

Among the antioxidative enzymes, SODs are responsible for the conversion of ${\text{O}}_{2}^{•-}$ to O_2_ and H_2_O_2_. Based on the metal present in the active center, these enzymes are classified as Cu/Zn-SOD (localized in the apoplast, cytosol, chloroplasts and peroxisomes), Mn-SOD (localized in mitochondria) or Fe-SOD (localized in chloroplasts) (Alscher et al., 2002; Das and Roychoudhury, 2014). Scavenging of H_2_O_2_ is performed by CAT, ascorbate peroxidase (APX), glutathione peroxidase (GPX), guaiacol peroxidase, class III peroxidases and peroxiredoxins. In general, peroxidases oxidize a wide range of substrates, thereby reducing peroxides including H_2_O_2_ (Mathé et al., 2010). While APX reduces H_2_O_2_ to H_2_O using the reducing power of AsA, GPX uses thioredoxin and GSH as electron donors (Das and Roychoudhury, 2014; Bela et al., 2015; Passaia and Margis-Pinheiro, 2015). On the other hand, guaiacol peroxidase prefers aromatic compounds such as guaiacol and pyrogallol as electron donors to reduce H_2_O_2_ (Das and Roychoudhury, 2014). As mentioned before, class III peroxidases can both scavenge and produce ROS. In their regular peroxidative cycle, they catalyze the reduction of H_2_O_2_ using a variety of electron donors including phenolic compounds, lignin precursors, secondary metabolites and auxins (Mathé et al., 2010; Zipor and Oren-Shamir, 2013). In contrast to the above-mentioned peroxidases, peroxiredoxins detoxify H_2_O_2_ by oxidizing their own thiol groups, which are back-reduced by the action of thioredoxin, glutaredoxin, cyclophilin or GSH (Tripathi et al., 2009). While GPX, guaiacol peroxidase, class III peroxidases and peroxiredoxins are also involved in other cellular processes, CAT and APX are specifically dedicated to H_2_O_2_ scavenging and the regulation of redox homeostasis. Therefore, both enzymes are discussed in more detail in this review (Table 1). Catalase is a tetrameric heme-containing enzyme catalyzing the detoxification of H_2_O_2_ to H_2_O and O_2_, which is mainly present in peroxisomes. The APX enzyme is localized in the cytosol, mitochondria, chloroplasts and peroxisomes and converts H_2_O_2_ into H_2_O during the first step of the AsA-GSH cycle (Das and Roychoudhury, 2014). While APX uses AsA as a reducing agent for H_2_O_2_ detoxification, the action of CAT does not require any reducing equivalents. This provides plants with an energy-efficient way of H_2_O_2_ removal, which can be of particular interest under stress conditions (Gechev et al., 2006). However, it is important to note that the affinity of APX for H_2_O_2_ is much higher than that of CAT (micromolar vs. millimolar range). Therefore, APX is assumed to be mainly involved in the fine-tuning of H_2_O_2_ detoxification important for its signaling function, while CAT is responsible for the bulk removal of excess H_2_O_2_ generated during stress conditions (Figure 2; Mittler, 2002). As shown in Table 1, both H_2_O_2_ scavenging enzymes are affected at transcriptional and activity level in metal-exposed plants. For example, Cuypers et al. (2011) demonstrated differential effects of Cd and Cu on *CAT* and *APX* gene expression in *Arabidopsis thaliana* plants. Dependent on the metal concentration and isoform considered, expression levels were specifically affected in roots or leaves. Furthermore, expression changes were not always mirrored by the enzyme activities, suggesting that CAT and APX regulation also occurs at the post-transcriptional level under metal stress (Cuypers et al., 2011).

### The link between H_2_O_2_ and metal tolerance in plants

In recent years, multiple studies have focused on the role of H_2_O_2_ in plant tolerance to a diverse array of abiotic stress conditions. Research has shown that pretreatment of plants with H_2_O_2_ can decrease the extent of adverse effects induced by subsequent exposure to abiotic stress factors including salinity, drought, heat, chilling and metals, a phenomenon which is generally referred to as H_2_O_2_ priming (Hossain et al., 2015). Exposure of plants to low concentrations of H_2_O_2_ (ranging from 100 to 500 μM) prior to metal treatment was shown to minimize metal-induced growth reduction, lipid peroxidation, chlorophyll degradation and programmed cell death in different plant species (Chao et al., 2009; Hu et al., 2009; Bai et al., 2011; Xu et al., 2011; Guzel and Terzi, 2013; Yildiz et al., 2013). Heat shock, known to increase H_2_O_2_ levels, can also induce metal tolerance in plants (Chao et al., 2009; Chou et al., 2012). Even though the mechanisms underlying these observations are not fully elucidated yet, available data so far point to the involvement of metal chelation, antioxidative defense and osmotic regulation in increased metal tolerance.

One of the key players in H_2_O_2_-induced metal tolerance is GSH. Indeed, many studies demonstrate an elevated GSH level in metal-exposed plants pretreated with H_2_O_2_ as compared to non-primed plants (Hu et al., 2009; Bai et al., 2011; Xu et al., 2011). As GSH is an important component of the AsA-GSH cycle, the elevated GSH level induced by H_2_O_2_ pretreatment of metal-exposed plants can contribute to an enhanced H_2_O_2_ detoxification, thereby reducing the negative effects of metal-induced oxidative stress (Apel and Hirt, 2004). Furthermore, GSH can directly chelate metals, which have a high affinity toward its sulfhydryl group. In addition, GSH is the main constituent of metal-chelating PCs. Metals sequestered by GSH and PCs are transported to the vacuole, decreasing the concentrations of free metal ions in the cytosol and thereby preventing metal-induced damage to cellular macromolecules such as DNA, proteins and membrane lipids. Moreover, vacuolar compartmentalization can also affect the transport of metals from roots to aerial plant parts (Liu W. J. et al., 2010; Jozefczak et al., 2012; Najmanova et al., 2012; Noctor et al., 2012). Indeed, Hu et al. (2009) and Bai et al. (2011) demonstrated a reduced root-to-shoot translocation of Cd in *O. sativa* plants pretreated with H_2_O_2_. In contrast, Yildiz et al. (2013) showed that H_2_O_2_ priming increased root-to-shoot translocation of Cr(VI) in *Brassica napus* plants. In these experiments however, H_2_O_2_ was able to counteract the decrease in fresh weight and the induction of lipid peroxidation caused by subsequent metal exposure. These data suggest that the mechanisms underlying H_2_O_2_-induced metal tolerance strongly depend on the metal and the plant species under study.

In addition to GSH, other antioxidants also seem to be involved in H_2_O_2_-induced metal tolerance. Xu et al. (2011) have shown that H_2_O_2_ priming enhanced the Al-induced increase in AsA levels in root tips of an Al-sensitive *Triticum aestivum* genotype. However, this was not observed in an Al-tolerant genotype, indicating that the inherent plant metal tolerance can influence the effect of exogenous H_2_O_2_ on the responses to subsequent metal exposure. Besides their levels, also the redox state of GSH and AsA can be affected, as indicated by increases in reduced vs. oxidized metabolite ratios by H_2_O_2_ priming in root tips of both *T. aestivum* genotypes after Al exposure (Xu et al., 2011).

Besides metabolic antioxidants such as GSH and AsA, also antioxidative enzymes could be involved in H_2_O_2_ priming. Indeed, several studies demonstrated differences in the activities of antioxidative enzymes such as SOD, CAT and APX between metal-exposed plants that were either primed with H_2_O_2_ or not (Chao et al., 2009; Hu et al., 2009; Xu et al., 2011; Yildiz et al., 2013). This is either related to the fact that H_2_O_2_ priming (1) counteracts a metal-induced reduction in antioxidative enzyme activities, probably due to binding of the metal to the protein's cysteine residues or (2) increases basal antioxidative enzyme activities to protect plants from metal-induced oxidative damage. Furthermore, it has been shown that H_2_O_2_ pretreatment can further stimulate metal-induced increases in the activity of GST (Hu et al., 2009; Bai et al., 2011). Together, these data suggest that H_2_O_2_ priming reduces the negative consequences of metal exposure, while stimulating the plant's defense mechanisms. This H_2_O_2_-induced enhancement of antioxidative defense, combined with an increase in metal scavenging, can possibly explain the fact that H_2_O_2_ priming often diminished metal-induced increases in ROS levels (Hu et al., 2009; Xu et al., 2011; Guzel and Terzi, 2013).

In addition to its effects on metal scavenging and antioxidative defense, other processes were also affected by H_2_O_2_ priming in metal-exposed plants. A study by Guzel and Terzi (2013) showed that H_2_O_2_ pretreatment counteracted the reductions in dry matter production, relative water content and water potential in leaves of Cu-exposed *Zea mays*. In addition, H_2_O_2_ priming reduced the negative effects of Cu on the levels of soluble proteins, sugars, and mineral ions and enhanced the Cu-mediated increase in proline content. These results suggest that the water balance may be a target of H_2_O_2_ priming in metal-exposed plants (Guzel and Terzi, 2013). Interestingly, proline levels are constitutively enhanced in different metal-tolerant plant species (Sharma and Dietz, 2006). While this may be related to its role in osmoregulation, proline might also confer metal tolerance through its function as metal chelator and ROS scavenger as discussed before (reviewed by Sharma and Dietz, 2006).

It is interesting to note that whereas H_2_O_2_ priming affects plant responses to metal stress, H_2_O_2_ alone (without subsequent metal exposure) does not always influence the parameters studied. As mentioned, metal-induced increases in antioxidative enzyme activities are often enhanced by H_2_O_2_ pretreatment. This does not always imply, however, that the activities of these enzymes are also increased in H_2_O_2_-primed plants that are not subsequently exposed to metal stress. In a recent review on this topic, Hossain et al. (2015) propose that pretreatment with H_2_O_2_ induces a mild oxidative challenge activating a ROS-dependent signaling network which results in the accumulation of latent defense proteins including antioxidative enzymes and transcription factors. As a consequence, plants enter a primed state that enables enhanced defense responses upon exposure to subsequent abiotic stressors such as metals.

It has been demonstrated that metal-induced oxidative stress is more powerful in sensitive genotypes or ecotypes (reviewed by Sharma and Dietz, 2009). Among the flowering plants, the metal hyperaccumulating plants *A. halleri, Noccaea caerulescens*, and *Alyssum bertolonii* exhibit a greater antioxidative capacity than their sensitive relatives (Sharma and Dietz, 2009). For example, activities of APX and class III peroxidases were highly increased in the Cd and Zn hyperaccumulator *A. halleri* as opposed to its sensitive counterpart *A. thaliana* (Chiang et al., 2006). In addition, CAT activity was more than 500 times higher in roots of the Ni hyperaccumulator *A. bertolonii* as compared to the non-hyperaccumulator *Nicotiana tabacum*, explaining the higher increase in H_2_O_2_ levels in the latter after Ni exposure (Boominathan and Doran, 2002). Interestingly, results of different studies on contrasting ecotypes or species indicate that H_2_O_2_ in particular is a crucial mediator of metal phytotoxicity. Indeed, tolerant and hyperaccumulating plant species often display a constitutively increased level of H_2_O_2_ scavenging enzymes (Sharma and Dietz, 2009). For example, Cho and Seo (2005) observed a higher survival rate and less lipid peroxidation in Cd-resistant *A. thaliana* mutants as compared to wild-type (WT) plants exposed to 300 or 500 μM Cd, even though the Cd content in the mutants was higher. The decreased Cd sensitivity of the mutants was mainly related to increased activities of several antioxidative enzymes such as APX and GR. Interestingly, the authors did not observe a relation between CAT activity and Cd tolerance. Nevertheless, Cd-resistant mutants had lower H_2_O_2_ levels as compared to WT plants (Cho and Seo, 2005), again supporting a role for H_2_O_2_ in plant metal tolerance. Furthermore, ROS production under metal stress could also mediate cross-tolerance to pathogens as reviewed by Poschenrieder et al. (2006). Underlying mechanisms could be the induction of antioxidants and the synthesis of secondary metabolites involved in mechanical defense against pathogen attack (Poschenrieder et al., 2006).

## Hydrogen peroxide mediates damage and/or signaling in metal-stressed plants

The balance between the generation and removal of ROS affects which reactive oxygen compound is present and at which level. This ultimately determines the extent of oxidative damage and/or signaling (Møller et al., 2007). Indeed, antioxidants function to limit the levels of ROS, thereby enabling them to execute beneficial cellular functions without causing too much damage (Halliwell, 2006). Based mainly on its concentration, but also on its production site and the plant's developmental stage, H_2_O_2_ affects plant stress responses in two ways (Petrov and Van Breusegem, 2012). In general, high levels of H_2_O_2_ induce cell death (Gechev and Hille, 2004; Petrov and Van Breusegem, 2012; Petrov et al., 2015). This process is critical during leaf senescence and the hypersensitive response, which are both known to occur in response to different developmental as well as environmental cues (Gechev et al., 2006; Quan et al., 2008; Petrov and Van Breusegem, 2012). At low concentrations, H_2_O_2_ acts as a signaling molecule by (1) directly affecting the activity of a target molecule involved in signaling or transcription, (2) oxidizing a biological molecule that in its turn acts as second messenger or (3) shifting the cellular redox balance to a more oxidized state (Apel and Hirt, 2004; Petrov and Van Breusegem, 2012). The essential role of H_2_O_2_ in cellular signaling is underlined by the global transcriptomic analysis of Desikan and coworkers, who demonstrated a H_2_O_2_-induced change in expression for approximately 1% of all *Arabidopsis* genes represented on the microarray (Desikan et al., 2001). In addition, H_2_O_2_ is a crucial mediator of plant responses to metal stress as discussed in the following sections.

Ample studies have demonstrated the occurrence of ROS-induced oxidative damage at the molecular level in plants exposed to various metals (Table 2). Lipids [especially polyunsaturated fatty acids (PUFAs)], DNA and proteins can be oxidatively damaged by ROS, depending on the reactivity of the latter. Hydrogen peroxide is moderately reactive as compared to other ROS and therefore only directly targets sulfur-containing residues in proteins (Møller et al., 2007). However, H_2_O_2_ can indirectly contribute to oxidative damage when it—together with ${\text{O}}_{2}^{•-}$—is converted to highly toxic ^•^OH radicals in the Fenton and Haber-Weiss reactions (Figure 1). Hydroxyl radicals are able to abstract a hydrogen atom from PUFA residues in a membrane, thereby initiating lipid peroxidation. The resulting carbon-centered radical quickly reacts with O_2_ to produce peroxyl radicals, attacking neighboring PUFA side chains and generating lipid hydroperoxides. These can freely decompose into different reactive species such as aldehydes (e.g. malondialdehyde) and lipid epoxides. Overall, lipid peroxidation leads to increased membrane leakiness and damage to receptors, enzymes and ion channels (Halliwell, 2006). Lipid peroxidation—concomitantly with a rise in H_2_O_2_/ROS levels—was shown to occur in different plant species exposed to Al (Pereira et al., 2010), Cd (Masood et al., 2012), Cu (Opdenakker et al., 2012a), Hg (Montero-Palmero et al., 2014), Ni (Khan and Khan, 2014), Pb (Kaur et al., 2015), and Zn (Khan and Khan, 2014; Table 2). It must be noted that redox-active metals accelerate lipid peroxidation by catalyzing the Fenton and Haber-Weiss reactions and splitting up lipid hydroperoxides into alkoxyl and new ^•^OH radicals to feed the chain reaction (Halliwell, 2006). This was clearly demonstrated by the results of Opdenakker et al. (2012a), comparing H_2_O_2_ levels and lipid peroxidation in *A. thaliana* plants exposed to either Cu or Cd in a similar setup. Both parameters were more rapidly increased and higher after exposure to the redox-active Cu as opposed to Cd, pointing toward a greater and quicker disturbance of the cellular redox state by the former metal (Opdenakker et al., 2012a). However, plant responses to specific metals must always be interpreted with the applied metal concentration, the duration of exposure, the cultivation system and the considered tissue(s) in mind. Interestingly, oxygenation of PUFAs leads to the production of oxylipins in an enzymatic or non-enzymatic manner (see Section “A Relationship between H_2_O_2_ and Oxylipins in Metal-Exposed Plants”). As oxylipins mediate plant responses to different stressors (Mithöfer et al., 2004; Dave and Graham, 2012), ROS-induced oxidation of lipids causes the emergence of new signaling molecules (Chmielowska-Bąk et al., 2015).

**Table 2:** **Oxidative damage in plants related to an elevated H_**2**_O_**2**_ content induced by metal exposure**.

**Metal**		**Species**	**Damage**						**References**
			**Molecular**				**Cellular**		
			**Lipid peroxidation**	**DNA damage**	**Protein oxidation**	**Hallmark genes**	**Chloroplast**	**Cell death**	
Essential	Cu	*Arabidopsis thaliana*	x						Cuypers et al., 2011
			x						Opdenakker et al., 2012a
			x				x		Martínez-Peñalver et al., 2012
		*Cucumis sativus*	x						İşeri et al., 2011
		*Hordeum vulgare*	x				x		Hu et al., 2015
		*Matricaria chamomilla*	x						Kováčik et al., 2010a,b
		*Medicago truncatula*		x	x		x		Macovei et al., 2010
		*Nicotiana tabacum*	x						Xia et al., 2012
		*Oryza sativa*	x				x		Mostofa et al., 2015a
			x						Thounaojam et al., 2012
		*Paulownia fortunei*	x				x		Wang J. et al., 2010
		*Solanum lycopersicum^a^*	x						İşeri et al., 2011
		*Spirodela polyrhiza*	x				x		Upadhyay and Panda, 2010
	Ni	*Brassica juncea*	x				x		Khan and Khan, 2014
		*Brassica napus*	x				x		Kazemi et al., 2010
		*Chlamydomonas reinhardtii*	x				x	x	Zheng et al., 2013
		*Vicia sativa*	x						Ivanishchev and Abramova, 2015
	Zn	*Brassica juncea*	x				x		Khan and Khan, 2014
		*Brassica napus*	x					x	Feigl et al., 2015
		*Brassica oleracea*	x						Barrameda-Medina et al., 2014
		*Lactuca sativa*	x						Barrameda-Medina et al., 2014
		*Myracrodruon urundeuva*	x						Gomes et al., 2013
		*Oryza sativa*	x						Thounaojam et al., 2012
		*Paulownia fortunei*	x				x		Wang J. et al., 2010
		*Phaseolus vulgaris*	x						Michael and Krishnaswamy, 2011
		*Populus × canescens*					x		Shi et al., 2015
		*Solanum melongena*	x						Wu et al., 2015
		*Solanum nigrum*	x					x	Xu J. et al., 2010
		*Spirodela polyrhiza*					x		Upadhyay and Panda, 2010
Non-essential	Al	*Cucumis sativus*	x		x		x		Pereira et al., 2010
		*Nicotiana tabacum*	x	x					Yin et al., 2010
		*Triticum aestivum*	x		x				Sun et al., 2015
	Cd	*Arabidopsis thaliana*	x						Cuypers et al., 2011
						x			Keunen et al., 2015
			x				x		Martínez-Peñalver et al., 2012
			x				x	x	Tao et al., 2013
Non-essential	Cd	*Boehmeria nivea*	x				x		Tang et al., 2015
		*Brassica campestris*	x						Anjum et al., 2014
		*Brassica juncea*	x				x		Masood et al., 2012
		*Brassica napus*	x				x		Ali et al., 2013
		*Citrus paradisi × Poncirus trifoliata*	x						Podazza et al., 2012
		*Dittrichia viscosa*	x				x		Fernández et al., 2013
		*Glycine max*	x		x				Pérez-Chaca et al., 2014
		*Helianthus annuus*	x						Saidi et al., 2014
		*Kosteletzkya virginica*	x		x		x		Han et al., 2013
		*Lactuca sativa*	x	x	x				Monteiro et al., 2012
		*Lepidium sativum*	x				x	x	Gill et al., 2012
		*Lupinus luteus*		x				x	Arasimowicz-Jelonek et al., 2012
		*Nicotiana tabacum*						x	Iannone et al., 2010
		*Oryza sativa*	x				x		Chou et al., 2011
			x				x		Mostofa et al., 2015b
			x					x	Singh and Shah, 2014
			x		x		x		Srivastava et al., 2014
			x		x			x	Srivastava et al., 2015
			x					x	Yu et al., 2015
		*Paulownia fortunei*	x				x		Wang J. et al., 2010
		*Populus cathayana*					x		He et al., 2013
		*Populus deltoides*					x		He et al., 2013
		*Populus × euramericana*					x		He et al., 2013
		*P. alba × P. glandulosa*					x		He et al., 2013
		*Sedum alfredii*	x						Tian et al., 2011
		*Solanum lycopersicum*	x				x		Ahammad et al., 2013
			x				x		Monteiro et al., 2011
		*Solanum nigrum*	x						Deng et al., 2010
								x	Liu et al., 2013
		*Trigonella foenum-graecum*	x				x		Zayneb et al., 2015
		*Triticum aestivum*	x				x		Moussa and El-Gamal, 2010
		*Vigna radiata*	x						Anjum et al., 2014
		*Zea mays*	x						Wahid and Khaliq, 2015
		*Zygophyllum fabago*	x				x		Yildiztugay and Ozfidan-Konakci, 2015
	Hg	*Juncus maritimus*	x		x				Anjum et al., 2015
		*Medicago sativa*	x						Montero-Palmero et al., 2014
	Pb	*Arabidopsis thaliana*	x				x	x	Tao et al., 2013
		*Atractylodes macrocephala*	x				x		Wang et al., 2013
Non-essential	Pb	*Brassica napus*	x						Ali et al., 2014
		*Hordeum vulgare*	x				x		Legocka et al., 2015
		*Nymphoides peltatum*	x				x		Qiao et al., 2013
		*Oryza sativa*	x		x		x		Srivastava et al., 2014
		*Paulownia fortunei*	x				x		Wang J. et al., 2010
		*Talinum triangulare*	x	x	x			x	Kumar et al., 2013
		*Triticum aestivum*	x					x	Kaur et al., 2013
			x						Kaur et al., 2015
		*Vicia faba*	x				x		Shahid et al., 2012
		*Zygophyllum fabago*	x				x		López-Orenes et al., 2014

a *In article as Lycopersicum esculentum*.

*Exposure to excess metals affects H_2_O_2_ production and causes molecular and cellular damage in plants. At the molecular level, lipids, DNA and proteins can be oxidized by H_2_O_2_. Expression of genes that are commonly induced by oxidative stress (Gadjev et al., 2006) can be assessed as marker of metal-induced oxidative damage. Furthermore, damage at the level of the chloroplast and even cell death are often observed under metal stress conditions. The effects of excess essential metals (Cu, Ni, and Zn) as well as non-essential metals (Al, Cd, Hg, and Pb) are shown and categorized based upon the metal and plant species studied. Only recently published papers (starting from 2010) demonstrating a metal-induced rise in H_2_O_2_ content and damage at molecular and/or cellular level were included in this overview*.

Although H_2_O_2_ itself is poorly reactive, different studies have demonstrated oxidative DNA damage and protein oxidation accompanied by an increased H_2_O_2_ level in various plant species under metal stress (Table 2). Oxidative DNA damage is often assessed by the amount of 8-hydroxyguanosine, the most commonly observed ROS-induced modification (Møller et al., 2007). Its levels were increased in Al-exposed *N. tabacum* (Yin et al., 2010) and Cu-treated *Medicago truncatula* plants (Macovei et al., 2010). Moreover, the alkaline comet assay revealed DNA damage in roots of Al-exposed *Allium cepa* (Achary et al., 2008), Cd-treated *Lactuca sativa* (Monteiro et al., 2012) and Pb-exposed *Talinum triangulare* plants (Kumar et al., 2013). Although many studies concentrated on DNA oxidation, it is now postulated that RNA is more susceptible to this process. Therefore, targeted RNA oxidation by ROS might be a novel mechanism to post-transcriptionally regulate expression of defense genes (Chmielowska-Bąk et al., 2015).

High intracellular levels of H_2_O_2_ oxidize both cysteine (-SH) and methionine (-SCH_3_) residues present in various proteins such as Cu/Zn- and Fe-SOD (Das and Roychoudhury, 2014). Although this may disrupt their enzymatic function and thereby lead to irreversible cell damage, it has been recently postulated to be a way to perceive and further relay a H_2_O_2_ signal in plant cells (Hardin et al., 2009; Petrov and Van Breusegem, 2012). In addition, protein carbonylation is commonly observed under metal stress (Table 2). For example, Al increased the carbonyl protein content in *A. cepa* roots (Achary et al., 2008) and *Cucumis sativus* seedlings (Pereira et al., 2010). Protein carbonyls were significantly enhanced in roots and leaves of *L. sativa* plants after Cd exposure (Monteiro et al., 2012), while similar results were observed in roots and shoots of *O. sativa* seedlings exposed to Pb (Srivastava et al., 2014). Not all proteins are equally sensitive to oxidation (Møller et al., 2007). For example, it has been demonstrated that mainly mitochondrial proteins are oxidized under well-irrigated and drought stress conditions in *T. aestivum* leaves (Bartoli et al., 2004). Moreover, Kristensen et al. (2004) have revealed specific subpopulations of *O. sativa* leaf mitochondrial matrix proteins that were carbonylated after *in vitro* treatment with H_2_O_2_ or Cu. Again, the possibility exists that ROS-mediated protein oxidation in plant mitochondria (and other compartments) functions as stress indicator, provoking an alarm signal to induce plant responses to developmental as well as environmental changes (Møller and Kristensen, 2004; Møller and Sweetlove, 2010; Chmielowska-Bąk et al., 2015). In conclusion, various oxidatively modified molecules serve as signaling compounds, supporting the view that oxidative damage and signaling are two sides of the same coin (Møller et al., 2007). Providing experimental evidence for this hypothesis during metal stress is an intriguing research challenge for the future.

In addition to damage at the molecular level, metal-exposed plants also suffer from (sub)cellular damage. This is often visible at the chloroplast level, leading to inhibition of photosynthesis (Table 2; Cuypers et al., 2009). Chlorophyll content was decreased in various plant species exposed to Al (Pereira et al., 2010), Cd (Zawoznik et al., 2007), Cu (Hu et al., 2015), Ni (Kazemi et al., 2010), Pb (Legocka et al., 2015), and Zn (Khan and Khan, 2014). In addition, different photosynthetic parameters (e.g. net photosynthesis rate) were reduced in *A. thaliana* plants exposed to Cd or Pb (Tao et al., 2013). Levels of H_2_O_2_ were significantly increased after metal exposure in all of the above-mentioned studies, pointing toward a correlation between H_2_O_2_ and the observed effects at the chlorophyll/photosynthesis level. In addition to chloroplast function and morphology, Cd exposure disturbed the distribution and mobility of mitochondria in *A. thaliana* protoplasts (Bi et al., 2009). Finally, it is important to note that metals are able to initiate H_2_O_2_-induced programmed cell death (Table 2). In Cd-exposed *N. tabacum* cells, NADPH oxidase was activated by a rise in cytosolic free Ca^2+^ concentrations, leading to H_2_O_2_ production and cell death (Garnier et al., 2006). Cadmium was also shown to increase the production of H_2_O_2_, which preceded cell death in *A. thaliana* cell suspension cultures (De Michele et al., 2009). Similarly, other studies indicate a relationship between metal exposure, oxidative stress and cell death using roots, root tips or leaf disks and different techniques to assess cell viability (Table 2; Pan et al., 2001; Achary et al., 2008; Iannone et al., 2010; Arasimowicz-Jelonek et al., 2012; Kumar et al., 2013; Feigl et al., 2015). Reactive oxygen species and H_2_O_2_ in particular are considered as crucial signals that modulate (programmed) cell death in plants (Gechev and Hille, 2004; Gadjev et al., 2008; Petrov et al., 2015), again highlighting the intimate relationship between ROS-mediated damage and signaling (Figure 2).

## Hydrogen peroxide directly mediates metal-induced oxidative signaling

The use of ROS as signaling molecules offers various potential advantages as discussed by Mittler et al. (2011). Their levels can rapidly change by shifting the balance between production and scavenging, which are both tightly controlled in space because of the presence of pro- and anti-oxidative enzymes at different subcellular locations (Mittler et al., 2004). The different molecular properties of various ROS offer the potential to transmit specific signals, also with regard to second messenger products formed after oxidative modification. Signaling is possible both within and across cells, generating a so-called ROS “wave” (Mittler et al., 2011; Baxter et al., 2014). Finally, ROS signaling integrates with several other signaling molecules and mechanisms such as Ca^2+^ and protein phosphorylation. In addition, ROS are directly linked to the plant's cellular homeostasis and metabolism. Therefore, they are perfectly suited to signal any metabolic change occurring during developmental and environmental stimuli (Mittler et al., 2011; Baxter et al., 2014).

Foyer and Noctor (2005) have described ROS-induced signaling through a “ripple” or domino effect over space and/or time, starting with a localized and/or transient oxidative burst affecting the expression of defense and regulatory genes in a transient or more sustained manner. Indeed, ROS are shown to activate various signaling compounds such as kinases/phosphatases, metabolites and hormones, which in their turn affect the expression of different target genes. This finally triggers acclimation to the altered developmental or environmental conditions a plant is experiencing (Mittler et al., 2004; Bienert and Chaumont, 2014). Particularly with regard to H_2_O_2_, it is interesting to note that it is produced in response to a wide variety of internal and external stimuli and therefore potentially contributes to cross-tolerance toward various stressors (Neill S. J. et al., 2002; Perez and Brown, 2014). Although oxidative stress commonly occurs in various stress conditions, the underlying signaling mechanisms may be highly stress-specific. This is underlined by the identification of marker transcripts specifically regulated by ^1^O_2_, ${\text{O}}_{2}^{•-}$ or H_2_O_2_ after exposure to different oxidative stress-causing agents. However, several transcripts were classified as general oxidative stress response markers because they responded to most of the applied treatments (Gadjev et al., 2006) and were also induced by Cd stress (Keunen et al., 2015; Table 2). Interestingly, Sewelam et al. (2014) have shown that H_2_O_2_ originating specifically from either chloroplasts or peroxisomes did have a differential impact on the *A. thaliana* transcriptome. Specificity of ROS-induced signaling might be related to the ROS type, amount, source and subcellular location of production, as well as their perception by different sensors (Miller et al., 2008; Cuypers et al., 2012).

### Perception of H_2_O_2_ during metal stress

Researchers have long been puzzled by the mechanism(s) used by plants to perceive stress-induced increases in H_2_O_2_ production and to relay this signal. A minimum of three potential mechanisms has been described: (1) H_2_O_2_ receptors that remain unidentified to date, (2) redox-sensitive transcription factors and (3) ROS-mediated inhibition of phosphatases (Mittler et al., 2004; Miller et al., 2008). Currently, it is still assumed that redox-sensitive transcription factors are oxidized by H_2_O_2_ and directly activate downstream signaling cascades (Neill S. et al., 2002; Miller and Mittler, 2006; Dietz, 2014). For example, class A heat shock factors (HSFs) are known to respond to oxidative stress in animals and plants (Petrov and Van Breusegem, 2012). The potential involvement of HSFs in perceiving H_2_O_2_ during metal stress (Miller and Mittler, 2006) is supported by the observed production of heat shock proteins in various metal-exposed plants (di Toppi and Gabbrielli, 1999; Cuypers et al., 2009). Miller et al. (2008) have proposed a model for ROS signaling using plants that lack the cytosolic APX1 isoform. In this model, different HSFs function as H_2_O_2_ sensors upstream of other transcription factors of the zinc finger protein ZAT (ZAT7, 10 and 12) and WRKY family (e.g. WRKY25) (Miller et al., 2008). Interestingly, expression levels of *ZAT12* and *WRKY25* genes were induced in *A. thaliana* plants exposed to Cd or Cu (Opdenakker et al., 2012a). Both genes were more rapidly induced upon exposure to Cu than to Cd in the roots, corresponding with the observed differences in H_2_O_2_ levels and potentially related to the contrasting redox properties of both metals (Opdenakker et al., 2012a).

A central protein involved in ROS sensing is the serine/threonine protein kinase oxidative signal-inducible 1 (OXI1). This enzyme is directly induced by H_2_O_2_ and forms an essential part of the signal transduction pathway linking ROS production to diverse downstream responses (Rentel et al., 2004). It also connects redox to lipid signaling via phosphatidic acid in a phosphoinositide-dependent kinase (PDK1)-related manner (Anthony et al., 2004, 2006). Interestingly, Opdenakker et al. (2012a) demonstrated highly increased *OXI1* transcription in Cd- or Cu-exposed *A. thaliana* plants. Again, its upregulation was higher and earlier induced after exposure to Cu, potentially related to its redox-active properties. Results by Smeets et al. (2013) underscore the key role of OXI1 in cellular signaling responses to Cu stress using *oxi1* knockout *A. thaliana* mutants. As compared to WT plants, plants lacking OXI1 responded differently to redox-induced changes (Smeets et al., 2013). Downstream of OXI1, mitogen-activated protein kinases (MAPKs) control the activation of multiple defense mechanisms in response to oxidative stress as discussed in the following section.

### Hydrogen peroxide signal transduction by MAPKs and transcription factors

One of the typical downstream signaling events associated with H_2_O_2_ sensing is the activation of MAPK pathways (Table 3; Mittler et al., 2004; Colcombet and Hirt, 2008). These signaling modules are found in all eukaryotic cells and consist of at least three kinases (MAP3K, MAP2K and MAPK) specifically phosphorylating and thereby activating each other (Colcombet and Hirt, 2008; Opdenakker et al., 2012b). Several authors have reported the involvement of MAPK signaling during exposure to Cd, Cu, Hg, Pb and Zn in different plant species (Opdenakker et al., 2012b and references therein). Upstream of MAPKs, the OXI1 kinase is considered to be a central player in metal-induced oxidative stress responses. Rentel et al. (2004) have shown that the activation of the MAPK isoforms MPK3 and MPK6 by H_2_O_2_ is reduced in *A. thaliana* plants lacking OXI1. Concurrently with *OXI1*, expression levels of its targets *MPK3* and *MPK6* were enhanced in Cd- or Cu-exposed *A. thaliana* plants (Opdenakker et al., 2012a). Jonak et al. (2004) studied the kinetics of different MAPK activities after exposure to either Cd or Cu in *M. sativa* seedlings. Similar to the results at the transcript level (Opdenakker et al., 2012a), Cu ions rapidly activated these enzymes while Cd exposure led to a delayed stimulation (Jonak et al., 2004). Since GSH effectively inhibited MPK3 and MPK6 activation in Cd-exposed *A. thaliana* plants, H_2_O_2_/ROS were shown to play a crucial role in this process (Liu X. M. et al., 2010).

**Table 3:** **Signaling responses related to an elevated H_**2**_O_**2**_ content induced by metal exposure**.

**Metal**		**Species**	**TFs**	**MAPKs**	**Phytohormones**	**References**
Essential	Cu	*Arabidopsis thaliana*	WRKY, ZAT	MPK3/6		Opdenakker et al., 2012a
					Aux	Yuan et al., 2013
		*Oryza sativa*			JA^a^	Mostofa et al., 2015a
		*Spirodela polyrhiza*			JA^a^	Upadhyay and Panda, 2010
	Ni	*Brassica juncea*			Eth	Khan and Khan, 2014
	Zn	*Brassica juncea*			Eth	Khan and Khan, 2014
		*Brassica oleracea*			JA^a^	Barrameda-Medina et al., 2014
		*Lactuca sativa*			JA^a^	Barrameda-Medina et al., 2014
		*Populus × canescens*			ABA, SA	Shi et al., 2015
		*Solanum melongena*			ABA, Aux, CK	Wu et al., 2015
Non-essential	Cd	*Arabidopsis thaliana*		MPK3/6		Liu X. M. et al., 2010
			WRKY, ZAT	MPK3/6		Opdenakker et al., 2012a
					JA^a^	Remans et al., 2010
					JA	Keunen et al., 2013
					SA	Tao et al., 2013
		*Brassica juncea*			Eth	Masood et al., 2012
		*Citrus paradisi* × *Poncirus trifoliata*			JA^a^	Podazza et al., 2012
		*Kosteletzkya virginica*			Aux, CK, Eth, SA	Han et al., 2013
		*Lupinus luteus*			SA	Arasimowicz-Jelonek et al., 2012
		*Oryza sativa*			JA^a^	Mostofa et al., 2015b
					Aux	Yu et al., 2015
		*Triticum aestivum*			ABA	Moussa and El-Gamal, 2010
	Hg	*Medicago sativa*			Eth	Montero-Palmero et al., 2014
	Pb	*Arabidopsis thaliana*			SA	Tao et al., 2013
		*Zygophyllum fabago*			SA	López-Orenes et al., 2014

a *Solely reported as an effect on LOX gene expression or LOX activity in article*.

*During metal stress, several signaling responses are induced by increased H_2_O_2_ levels. Several transcription factors (TFs) and MAPKs and are activated by H_2_O_2_. In addition, multiple phytohormone signaling pathways are affected by different metals. The effects of excess essential metals (Cu, Ni, and Zn) as well as non-essential metals (Al, Cd, Hg, and Pb) are shown and categorized based upon the metal and plant species studied. Only recently published papers (starting from 2010) demonstrating a metal-induced rise in H_2_O_2_ content and signaling were included in this overview. Abbreviations: ABA, abscisic acid; Aux, auxins; CK, cytokinin; Eth, ethylene; JA, jasmonic acid; SA, salicylic acid*.

In addition to OXI1, also the MAP3K *Arabidopsis* NPK1-like protein kinase 1 (ANP1) is directly activated by H_2_O_2_ and initiates a phosphorylation cascade via MPK3 and MPK6 (Kovtun et al., 2000). Expression levels of *ANP1* were increased in roots of Cu-exposed *A. thaliana* plants after 6 and 24 h (Opdenakker et al., 2012a). Although MAPKs can be activated by H_2_O_2_, they also trigger an H_2_O_2_-mediated oxidative burst themselves (Mittler et al., 2004; Petrov and Van Breusegem, 2012). Indeed, MEK2 (the *Nicotiana* ortholog of *Arabidopsis* MKK4/5) was implicated in ROS production upon fungal infection in *N. benthamiana* by acting upstream of *RBOH* genes known to evoke H_2_O_2_ production (Yoshioka et al., 2003). Similarly, expression of constitutively active MKK4/5 led to H_2_O_2_ generation and cell death in *A. thaliana* (Ren et al., 2002). As MAPK cascades function both up- and downstream of H_2_O_2_ (Mittler et al., 2004; Pitzschke and Hirt, 2006; Pitzschke et al., 2009; Petrov and Van Breusegem, 2012), the existence of positive feedback loops between H_2_O_2_ and MAPKs such as MKK4/5 deserves further attention under metal stress conditions.

Activated MAPK cascades are able to regulate downstream gene expression by activating or repressing transcription factors (Colcombet and Hirt, 2008). Transcription factors of the ZAT, WRKY, NAC, DREB, bZIP and MYB family therefore constitute the final link in the signaling chain induced by H_2_O_2_ (Petrov and Van Breusegem, 2012). Results by Pitzschke et al. (2009) have demonstrated the involvement of a complete MAPK cascade consisting of MEKK1, MKK1/MKK2, and MPK4 in regulating ROS-induced stress signaling. Indeed, the majority of transcription factors responsive to multiple ROS-producing conditions are controlled by this pathway (Pitzschke et al., 2009). Furthermore, MEKK1 is able to directly interact with and phosphorylate the transcription factor WRKY53 (Miao et al., 2007), which could be involved in metal-induced senescence (see Section “Metal-Induced Responses at the Cellular Level: is H_2_O_2_ Involved in Root Growth Inhibition and Senescence?”).

Different members of the ZAT family of zinc finger transcription factors were strongly induced by ROS at the transcript level (Gadjev et al., 2006). In particular, isoforms 7, 10 and 12 have been put forward to be involved in ROS signaling during abiotic stress (Davletova et al., 2005a; Miller et al., 2008). In addition, WRKY transcription factors could function up- or downstream of ZAT proteins (Miller et al., 2008). The WRKY proteins, belonging to one of the largest transcription factor families in plants (Eulgem and Somssich, 2007), all contain the invariable WRKY amino acid signature and recognize W-box *cis* elements in target gene promoter regions. The induction of WRKY25 during oxidative stress was shown to be ZAT12-dependent (Rizhsky et al., 2004). As mentioned before, both *ZAT12* and *WRKY25* expression was induced in Cd- or Cu-exposed *A. thaliana* plants (Opdenakker et al., 2012a), further supporting their involvement in metal-induced ROS signaling. For members of the NAC, DREB, bZIP and MYB family associated with H_2_O_2_ signaling, their relation to metal stress is to our knowledge generally unexplored to date. Nevertheless, several NAC transcription factors were shown to be H_2_O_2_-responsive (Balazadeh et al., 2010) and govern leaf senescence in *A. thaliana* (Balazadeh et al., 2008). As discussed in the Section “Metal-Induced Responses at the Cellular Level: Is H_2_O_2_ Involved in Root Growth Inhibition and Senescence?,” metal exposure might induce a hastening of this naturally occurring process and the role of NAC transcription factors herein might be an interesting topic for future research. This is further supported by promising results of Fang and coworkers, who recently demonstrated the stress-responsive SNAC3 transcription factor to confer tolerance to heat and drought stress in *O. sativa* plants by modulating ROS (Fang et al., 2015).

Although OXI1, MPK3 and MPK6 were shown to be activated in metal-exposed plants, information on upstream signaling pathways as well as downstream targets under metal stress conditions is rather scarce. Nevertheless, defined end points of specific MAPK signaling pathways are critical to activate the plant's antioxidative defense during metal-induced oxidative stress (Cuypers et al., 2012). In response to H_2_O_2_, MAPK regulation of ZAT12 led to enhanced expression of the *APX1* gene in *A. thaliana* (Rizhsky et al., 2004). This gene, encoding a cytosolic H_2_O_2_ scavenging enzyme, was shown to protect the chloroplast redox state during light stress (Davletova et al., 2005b). Interestingly, also the *CAT1* gene was shown to be regulated by MAPK signaling in *A. thaliana* (Xing et al., 2007, 2008). Both *APX1* and *CAT1* are critical in scavenging metal-induced H_2_O_2_ and were induced in *A. thaliana* plants exposed to Cd, Cu, or Zn (Table 1; Cuypers et al., 2011; Remans et al., 2012a). Interestingly, Davletova et al. (2005b) have postulated the involvement of MAPK-regulated *RBOHD* expression in ROS signal amplification during light stress, and further studies confirmed its role in abiotic stress-induced systemic signaling (Miller et al., 2009). Expression of *RBOHD* was also induced upon Cd, Cu and Zn exposure in *A. thaliana* (Remans et al., 2010, 2012a; Cuypers et al., 2011). Although all of the above-mentioned components have been separately assessed under metal stress conditions, further efforts should be made to reveal the sequence of events from stress perception to response in metal-exposed plants.

Metal-induced MAPK signaling pathways show extensive crosstalk with phytohormone signaling. Upon activation, both MPK3 and MPK6 can phosphorylate 1-aminocyclopropane-1-carboxylate synthase (ACS) isoforms 2 and 6, increasing their half-life and the production of ethylene by these enzymes (Liu and Zhang, 2004; Joo et al., 2008; Han et al., 2010). Transcription of both ACS isoforms can also be enhanced by MPK3/6 via the WRKY33 transcription factor (Li et al., 2012). In addition, Yoo et al. (2008) have shown that a MKK9-MPK3/6 cascade promotes ethylene signaling by phosphorylating the nuclear transcription factor ethylene-insensitive 3 (EIN3) in *A. thaliana*. Increasing evidence supports a role for ethylene in regulating metal stress responses in plants (reviewed by Thao et al., 2015; Keunen et al., 2016). It has been demonstrated that the increase in ethylene levels was mainly related to upregulated *ACS2* and *ACS6* expression in Cd-exposed *A. thaliana* plants (Schellingen et al., 2014). Furthermore, MPK3 and MPK6 were proposed to connect ROS production to ethylene signaling in *A. thaliana* leaves under Cd exposure. Cadmium activates NADPH oxidases that produce ROS, which are sensed by OXI1. This kinase then activates MPK3 and MPK6, both affecting ACS2 and ACS6 enzymes at various levels (Schellingen et al., 2015). In conclusion, ethylene shows extensive crosstalk with signaling by ROS or H_2_O_2_ under metal stress (Thao et al., 2015; Keunen et al., 2016), which should definitely be explored in more detail in future studies. Also the production of other phytohormones such as abscisic acid (ABA), auxins, cytokinins, jasmonic acid (JA) and salicylic acid (SA) is affected by metal exposure in different plant species (Table 3). Compelling evidence for a role of endogenous SA in Pb and Cd tolerance of *A. thaliana* was provided by Tao et al. (2013). Metal-induced phytotoxicity was potentiated by elevating endogenous SA levels, while plants with lower SA levels performed better when exposed to Pb or Cd. One of the underlying mechanisms of SA-mediated toxicity is related to plant redox homeostasis, with SA-accumulating plants showing higher metal-induced H_2_O_2_ concentrations as compared to SA-deficient plants (Tao et al., 2013). As discussed by Petrov and Van Breusegem (2012), interactions between H_2_O_2_ and SA can range from cooperation to inhibition depending on the used experimental conditions. Therefore, much work remains to be done to fully unravel the interaction between H_2_O_2_ and phytohormones such as ethylene and SA during metal stress in plants. In addition, a link between H_2_O_2_ and JA in metal-exposed plants is evident and discussed in the Section “A Relationship between H_2_O_2_ and Oxylipins in Metal-Exposed Plants”.

## Hydrogen peroxide interacts with other signaling pathways and regulating mechanisms

As mentioned before, H_2_O_2_ is connected to a variety of signaling molecules (e.g. MAPK) and plant hormones (e.g. ethylene). In this section, we discuss its relation to Ca^2+^, nitric oxide (NO^•^), oxylipins and microRNAs in general and demonstrate evidence for their involvement during the metal-induced oxidative challenge in plants (Figure 2).

### Interaction between H_2_O_2_ and Ca^2+^ in metal-exposed plants

Compelling evidence indicates a reciprocal relationship between H_2_O_2_ and Ca^2+^, two crucial messengers involved in plant responses to multiple stress conditions (Tuteja and Mahajan, 2007; Quan et al., 2008; Mazars et al., 2010; Petrov and Van Breusegem, 2012). Rentel and Knight (2004) observed a biphasic increase in cytosolic Ca^2+^ levels of *Arabidopsis* seedlings upon treatment with H_2_O_2_. Enhancing or reducing the height of the Ca^2+^ peaks had a corresponding effect on the expression of the H_2_O_2_-responsive *GST1* gene, indicating crosstalk between H_2_O_2_ and Ca^2+^ signaling in plants (Rentel and Knight, 2004). Whereas ROS modulate cytosolic Ca^2+^ levels through the activation of Ca^2+^ channels in the plasma membrane, H_2_O_2_ production by NADPH oxidases reversely depends on Ca^2+^ (reviewed by Mazars et al., 2010). In Cd-exposed bright yellow-2 *N. tabacum* cells, H_2_O_2_ production was preceded by an enhanced cytosolic Ca^2+^ level essential to activate NADPH oxidases (Garnier et al., 2006). Indeed, Ca^2+^ directly binds EF-hand motifs in the cytosolic N-terminal domain of the NADPH oxidase enzyme and leads to phosphorylation of the N-terminus by activating a calcium-dependent protein kinase (CDPK) (Sagi and Fluhr, 2006; Kobayashi et al., 2007; Ogasawara et al., 2008). The potential involvement of CDPK in metal stress responses is supported by the transcriptional induction of the *CDPK1* gene in roots of Cd-exposed *A. thaliana* plants (Smeets et al., 2013). Furthermore, several CDPK isoforms in *T. aestivum* were responsive to H_2_O_2_ treatment, indicating a role for these enzymes in oxidative signaling in plants (Li et al., 2008; Schulz et al., 2013). Interestingly, an increased Ca^2+^ concentration in peroxisomes caused by elevated cytosolic Ca^2+^ levels was shown to stimulate CAT3 activity *in vivo*. The resulting rise in peroxisomal H_2_O_2_ scavenging potential (Costa et al., 2010) could also be important during metal-induced oxidative stress. In this regard, the cellular response of *Pisum sativum* plants to long-term Cd exposure was shown to involve extensive crosstalk between Ca^2+^, ROS and NO^•^ (Rodríguez-Serrano et al., 2009) as discussed in the following section. Finally, Baliardini et al. (2015) recently reported a positive correlation between the expression of a gene encoding a Ca^2+^/H^+^ exchanger (*CAX1*) and Cd tolerance in *Arabidopsis*. Indeed, its expression was higher in the Cd-tolerant *A. halleri* as compared to its Cd-sensitive relative species *A. lyrata* and *A. thaliana*. Plants without functional CAX1 also show increased accumulation of H_2_O_2_ when exposed to Cd, suggesting a role for CAX1 in maintaining cytosolic Ca^2+^ levels and thereby avoid uncontrolled ROS accumulation during oxidative stress conditions (Baliardini et al., 2015).

### Nitric oxide and H_2_O_2_: friends or foes during metal exposure?

Nitric oxide (NO^•^) production is often induced by abiotic stress in plants, for example during exposure to different metals (reviewed by Xiong et al., 2010). In contrast, *P. sativum* plants showed reduced NO^•^ levels under long-term (14 days) Cd exposure (Rodríguez-Serrano et al., 2009). The authors hypothesized, since NO^•^ is able to react with ${\text{O}}_{2}^{•-}$, that these lower NO^•^ levels could result in ${\text{O}}_{2}^{•-}$ accumulation under Cd stress. This was further supported by decreased ${\text{O}}_{2}^{•-}$ levels when NO^•^ production was restored in Cd-exposed plants by application of additional Ca (Rodríguez-Serrano et al., 2009). Different authors have reported the potential of exogenous NO^•^ to alleviate metal toxicity in plants (Xiong et al., 2010). For example, it has been proposed that NO^•^-induced Cu tolerance in *Lycopersicon esculentum* plants was mediated by H_2_O_2_ detoxification and the accumulation of Cu-scavenging metallothioneins (Wang L. et al., 2010). Although external application of NO^•^ activated the antioxidative defense system, endogenous NO^•^ could also contribute to metal phytotoxicity (reviewed by Arasimowicz-Jelonek et al., 2011). For example, NO^•^ is known to promote the upregulation of genes involved in Fe uptake under Cd stress, thereby also contributing to increased Cd uptake in *A. thaliana* (Besson-Bard and Wendehenne, 2009; Besson-Bard et al., 2009). On the other hand, it is proposed that NO^•^ produced by plants challenged with low Cd concentrations could mediate signaling responses leading toward metal tolerance (Arasimowicz-Jelonek et al., 2011). It is clear that further research is required to fully unravel the role of NO^•^ and its interaction with H_2_O_2_ and oxidative stress (Petrov and Van Breusegem, 2012) during metal exposure in plants.

### A relationship between H_2_O_2_ and oxylipins in metal-exposed plants

Various stress stimuli, such as exposure to different metals, activate biosynthetic enzymes responsible for the accumulation of oxylipins. These are derived from the oxidation of PUFAs by lipoxygenase (LOX) enzymes, with the phytohormone JA and its volatile derivative methyl jasmonate (MeJA) often considered to be the most important in signaling (Browse, 2009; Dave and Graham, 2012; Santino et al., 2013; Wasternack and Hause, 2013). In addition, a non-enzymatic route triggered by ROS is responsible for the synthesis of phytoprostane oxylipins that are also involved in plant stress responses (Dave and Graham, 2012). Evidence for a role of oxylipins during metal stress is provided by the observed induction of LOX at the transcript and activity level in various plant species (Table 3; Skórzyńska-Polit et al., 2006; Tamás et al., 2009; Remans et al., 2010; Keunen et al., 2013; Barrameda-Medina et al., 2014). Furthermore, JA levels increased in *A. thaliana* and *Phaseolus coccineus* plants exposed to Cd or Cu (Maksymiec et al., 2005), supporting a role for JA signaling in mediating stress responses in metal-exposed plants (Maksymiec, 2007). For example, MeJA was shown to upregulate the same set of genes involved in GSH biosynthesis that were also induced in Cd- or Cu-exposed *A. thaliana* plants (Xiang and Oliver, 1998). Interestingly, exogenously applied MeJA induced H_2_O_2_ production, lipid peroxidation and LOX activity in *Taxus chinensis* cells (Wang and Wu, 2005). Similarly, application of MeJA to *A. thaliana* roots strongly increased H_2_O_2_ concentrations in the leaves (Maksymiec and Krupa, 2002). This points toward a link between both JA and H_2_O_2_, suggesting that JA may contribute to metal-induced oxidative stress responses in plants (Rodríguez-Serrano et al., 2009).

### MicroRNAs and redox signaling in metal-exposed plants

Together with small interfering RNAs (siRNAs), microRNAs (miRNAs) are endogenous non-coding small RNAs involved in the regulation of plant development and stress responses (Vazquez et al., 2010). MicroRNAs negatively regulate their target genes by (1) mRNA cleavage or inhibition of translation or (2) DNA methylation. Expression of different miRNAs is affected by metal stress in different plant species (reviewed by Gielen et al., 2012; Gupta et al., 2014). In general, miRNA-mediated responses are related to metal complexation, antioxidative defense and stress signaling. For example, miR395 regulates sulfate assimilation and was induced in Cd-exposed *B. napus* seedlings (Huang et al., 2010). Sulfate assimilation into cysteine is ultimately required to synthesize GSH and PCs able to chelate free Cd ions, suggesting a role for miR395 in regulating Cd complexation in plants (Gielen et al., 2012). In *Arabidopsis, miR398* expression is downregulated by excess Cu, resulting in transcriptional induction of its target genes *Cu/Zn-SOD 1* and *2* (*CSD1/*2). As compared to Cu, Cd exposure oppositely affected both *miRNA398* and *CSD1/2* expression levels, indicating metal-specific regulation potentially related to the redox-active vs. non-redox-active nature of Cu vs. Cd (Cuypers et al., 2011). Interestingly, Cu exposure did not reduce *miR398* expression in leaves of *A. thaliana* plants lacking functional OXI1 as it did in WT plants, pointing toward an interaction between miR398 and MAPK signaling during metal stress (Smeets et al., 2013). Finally, various target genes of metal-induced miRNAs are involved in phytohormone biosynthesis and signaling, often by affecting transcription factors (Gielen et al., 2012; Gupta et al., 2014). Panda and Sunkar (2015) have recently discussed the potential role of redox signaling and/or ROS in inducing stress-responsive miRNAs in plants. This is further supported by a genome-wide study in *O. sativa*, showing seven miRNA families to be induced or downregulated by H_2_O_2_ treatment (Li et al., 2011). One of the miRNAs upregulated by H_2_O_2_ is *miR397*, targeting laccase enzymes involved in lignin biosynthesis. Interestingly, metal exposure was also shown to induce *miR397* (reviewed by Gielen et al., 2012; Gupta et al., 2014), suggesting a potential role for H_2_O_2_ in mediating this induction under metal stress conditions. Future studies should aim to unravel the interplay between metal-induced production of ROS/H_2_O_2_ and its effects on the induction or downregulation of specific miRNAs targeting downstream response genes.

## Metal-induced responses at the cellular level: is H_2_O_2_ involved in root growth inhibition and senescence?

As indicated in Tables 1–3, metal exposure increases H_2_O_2_ levels in a variety of plant species, thereby inducing both oxidative damage and signaling responses. At the cellular level, this might underlie metal-induced responses observed in roots (e.g. growth inhibition) and leaves (e.g. premature senescence). For example, Cd-induced oxidative stress could be related to the inhibition of root initiation and elongation (Lux et al., 2011). However, also plant hormones might regulate root growth of metal-exposed plants (Remans et al., 2012b; De Smet et al., 2015). As ROS are shown to interact with phytohormones such as ethylene, future research efforts should be made to dissect their role as potential modulators of root development under metal stress conditions.

Many of the parameters listed in Table 2 (e.g. lipid peroxidation) can also be regarded as indicators of plant senescence. Indeed, it is known that plants exposed to metals such as Cu and Cd show an accelerated appearance of senescence symptoms (Maksymiec, 2007). During the senescence process, leaves are degraded in a highly regulated fashion in order to remobilize nutrients to developing plant tissues. Leaf senescence comprises the final stage of leaf development and its onset is determined by the developmental age of leaves (Lim et al., 2007). It has been shown, however, that this process can be prematurely induced by several biotic and abiotic stress factors such as pathogen attack, wounding, darkness, drought, salinity, UV-B irradiation and ozone (Miller et al., 1999; John et al., 2001; Espinoza et al., 2007; Zhou et al., 2011; Guo and Gan, 2012; Allu et al., 2014; Zhou et al., 2014).

An important characteristic of senescence is the degradation of cellular macromolecules such as chlorophyll, lipids, proteins and nucleic acids. During the end stage of senescence, cells undergo programmed cell death (Lim et al., 2007). As shown in Table 2, many of these features are also affected by metal exposure in plants. In addition, it is known that several components of metal-induced signaling responses are also key players in the initiation and regulation of the senescence process. For example, changes in phytohormone levels are known to affect the onset of leaf senescence. While cytokinins, gibberellins and auxins delay the appearance of senescence symptoms, increases in the levels of other phytohormones such as ethylene, ABA, JA and SA have been shown to accelerate the process (Lim et al., 2007; Fischer, 2012).

Furthermore, transcriptional regulation mechanisms also play an important role in leaf senescence. In *A. thaliana* leaves, for example, more than 800 genes are differentially expressed during senescence (Buchanan-Wollaston et al., 2005). While certain genes such as those encoding photosynthetic proteins are transcriptionally downregulated, the expression of many other genes significantly increases when leaves enter the senescent stage. The latter genes are generally termed “senescence-associated genes” or SAGs and encode proteins involved in the breakdown of cellular compounds (e.g. nucleases, proteases and cell wall hydrolases) and the remobilization of nutrients to developing plant tissues. Also numerous transcription factors, many of which belong to the NAC and WRKY transcription factor families, are considered as SAGs (Miao et al., 2004; Fischer, 2012). For example, overexpression of the NAC transcription factor ORESARA1 SISTER1 (ORS1) accelerates senescence in *A. thaliana*, whereas the appearance of senescence symptoms is delayed in plants lacking functional ORS1. Furthermore, 42 genes were shown to be induced by ORS1, many of which are known to be involved in age-dependent senescence and in the response to long-term salinity (Balazadeh et al., 2011). Of the WRKY transcription factors, *WRKY53* is one of the most studied genes with regard to senescence. It can affect the expression of several other transcription factors including other WRKYs, indicating that it might be a key player in a transcription factor signaling cascade (Miao et al., 2004). In addition, the MAP3K MEKK1 can directly phosphorylate the WRKY53 protein thereby increasing its DNA-binding activity, suggesting that MAPK signaling is also involved in the regulation of senescence (Miao et al., 2007). This idea is supported by the fact that plants overexpressing or lacking MKK9 and MPK6 show an accelerated or delayed onset of senescence, respectively (Zhou et al., 2009).

As mentioned above, metal exposure induces many effects associated with senescence in a broad range of plant species (Table 2). McCarthy et al. (2001) demonstrated Cd-induced increases in lipid peroxidation and protease activity in *P. sativum* leaves. Furthermore, they reported a decreased leaf chlorophyll content and a disorganization of chloroplast structure in leaves of Cd-exposed plants. Similar results were obtained by Rodríguez-Serrano et al. (2006), showing Cd-induced lipid peroxidation in *P. sativum* roots. In addition, levels of the senescence-promoting phytohormones SA, JA and ethylene were significantly elevated in roots of Cd-exposed plants as compared to those of control plants. Interestingly, these changes were accompanied by increases in ${\text{O}}_{2}^{•-}$ and H_2_O_2_ levels, suggesting a role for ROS in Cd-induced accelerated senescence. In addition to Cd, other metals were shown to induce senescence-associated processes as well. Upadhyay and Panda (2010) demonstrated lipid peroxidation and decreased chlorophyll content associated with increased ROS levels in *Spirodela polyrhiza*. Furthermore, lipid peroxidation and negative effects on chlorophyll content or chloroplast structure were reported in Pb-exposed *Ceratophyllum demersum* (Mishra et al., 2006) and Zn-exposed *Hydrilla verticillata* (Xu et al., 2013).

Taken together, these data strongly suggest that metal exposure induces accelerated senescence in plants. However, little or no data are available on the effect of metal exposure on SAG expression levels. It is known, however, that transcription of many SAGs is increased in plants treated with H_2_O_2_ (Miao et al., 2004; Yan et al., 2007; Zhou et al., 2013, Zhou et al., 2014). Interestingly, *ORS1* and *WRKY53* expression was also induced by H_2_O_2_, suggesting that both transcription factors play a key role in the H_2_O_2_-induced senescence response in plants (Miao et al., 2004; Balazadeh et al., 2011).

A role for ROS in regulating senescence is further supported by the observed increased concentrations of ${\text{O}}_{2}^{•-}$ and H_2_O_2_ in senescing tissues (Fischer, 2012). This can be caused by lipid peroxidation, which is known to occur during senescence (Zimmermann and Zentgraf, 2005). However, it could also be due to a decrease in the plant's antioxidative defense as reported by several authors (Jiménez et al., 1998; Prochazkova et al., 2001; Procházková and Wilhelmová, 2007). This hypothesis is further supported by the fact that the *Arabidopsis vtc1-1* mutant, which is deficient in the antioxidative metabolite AsA, has a higher expression of certain SAGs and an earlier appearance of senescence symptoms as compared to WT plants (Barth et al., 2004). In addition to AsA, also the antioxidative enzyme CAT could be involved in regulating senescence. Indeed, Zimmermann et al. (2006) proposed that a downregulation of the *CAT2* isoform contributes to the senescence-associated H_2_O_2_ peak, subsequently causing an increase in the expression levels of the stress-responsive *CAT3* gene. Interestingly, Cuypers et al. (2011) reported a downregulation of *CAT2* and an upregulation of *CAT3* in Cd-exposed *A. thaliana* plants, possibly pointing to a Cd-induced acceleration of senescence.

As metals are known to increase ROS production, thereby inducing an oxidative challenge, we hypothesize a role for H_2_O_2_ in the damage and signaling events ultimately leading to premature leaf senescence under metal stress. In order to gain more insight into the effect of metal exposure on leaf senescence, future research should aim to identify the influence of different metals on the expression levels of SAGs including transcription factors such as ORS1 and WRKY53.

## Conclusions and a look forward

By compiling the gathered evidence, the role of ROS and particularly H_2_O_2_ in regulating metal stress responses in plants is unequivocally demonstrated. Furthermore, it is becoming increasingly clear that oxidative damage and signaling are two sides of the same coin, potentially cooperating to establish plant acclimation and tolerance to metal exposure. Different studies highlight the interaction between ROS/H_2_O_2_ and signaling components such as MAPKs, phytohormones, Ca^2+^, NO^•^, oxylipins and regulating systems like miRNAs (Figure 2). Nevertheless, our current knowledge only represents the tip of the iceberg, encouraging further research efforts in the field of H_2_O_2_ perception, signal transduction and its role in plant acclimation to and growth under metal stress conditions.

## Author contributions

All authors participated in the conception of the topic. AC, SH and EK wrote the manuscript. Figures and Tables were designed by RAR, SDS, JD, HG, MJ, CL and HV. All authors read and approved the final manuscript after critically revising it for important intellectual content.

## Funding

This work was supported by the Research Foundation Flanders (FWO) by a postdoctoral grant for EK and projects [G0D3414] and [G0D1114]. Additional funding came from Hasselt University (BOF12NI28, BOF14DOC04) and PhD grants from the Agency for Innovation by Science and Technology (IWT-Flanders).

### Conflict of interest statement

The authors declare that the research was conducted in the absence of any commercial or financial relationships that could be construed as a potential conflict of interest.
